# Venereal Disease in the Army

**Published:** 1905-11-11

**Authors:** 


### VENEREAL DISEASE IN THE ARMY.

Major T. McCulloch, R.A.M.C.,[1] has recently published a collection of facts dealing with this highly important matter. The contribution is exceedingly valuable to all serious students of an important national problem, because it contains much of its information in the handy form of tabulated figures, or of curves representing the yearly frequency of the different items in the venereal catalogue over a considerable series of years.

In the first place, it appears that the rejections on account of syphilis among applicants for enlistment have fallen considerably since the year 1883. Previously to that year these had varied from eleven to sixteen per thousand; but in 1883 there occurred a sudden drop to 9.8 per thousand, and since then the figure has been steadily lessening. This very curious fact does not appear capable of a completely satisfactory explanation. Major McCulloch notes that the fall in the rejections for venereal disease coincided in point of time with the inauguration of the short service system, and suggests that the prospects of short service tempted to the service a class of man somewhat more likely to be self-controlled than did the earlier regulations.

As regards the army in India, the percentage of admissions to hospital on account of venereal diseases reached their minimum in about the year 1873, and thenceforward mounted gradually, reaching their maximum in 1895, when admissions for such diseases achieved the appalling total of 536.9 per thousand. From this year onward the figure fell again, being reduced by 1903 to 249.5 per thousand. It is worthy of notice that the general increase in the venereal admissions among the army in India again coincided with the institution of short service, and depended in part, no doubt, upon the increased circulation of men.

But while the frequency of venereal disease among soldiers in India is seen to have increased in the way described above, it has fallen for the army at home. Moreover, the type of syphilis common to India is far more severe than that usually seen at home. This is evidenced by the following statement of the invaliding on account of syphilis during the ten years following 1894. During this interval there were invalided from all foreign stations 4,378 men, of which number India supplied not less than 3,792; and of 2,875 men discharged unfit for service through syphilis, no less than 1,413 were invalids from the Indian army.

Commenting upon the coincidence of an increase in the invaliding for venereal diseases in India with a decrease at home, the author considers the determining factors in the production of such a state of things to be as follows. The young soldier recently enrolled at home is generally untrained, and therefore has his time fully occupied with the duties of his apprenticeship, and is, moreover, financially in a depressed condition. Presently he is drafted for foreign service, where, being fully trained, he has more time and money at his disposal, and lacks besides the home influence, which, however indifferently, undoubtedly does to some extent counteract the temptations of barrack life.

Taking the home and foreign services together, there appears to be a gratifying decrease in the invaliding from syphilis. This is credited by the author to the better education of the soldier, to the provision of sports and recreations of various kinds, both outdoor and indoor, to the provision of libraries, and to the institution of health lectures. The author expresses no strong views about the much-criticised Contagious Diseases Act, but quotes facts from the official returns which appear to demonstrate that this measure did not have the good effect which is often claimed for it.

[1] Jour. of R.A.M.C., October, 1905.

